# Supplementary figures and images for: New Diagnostic and Prognostic Models for the Development of Alcoholic Cirrhosis Based on Genetic Predisposition and Alcohol History
Source: Biomedicines. 2023 Jul 28;11(8):2132. doi: 10.3390/biomedicines11082132 (PMC10452718; doi:10.3390/biomedicines11082132)

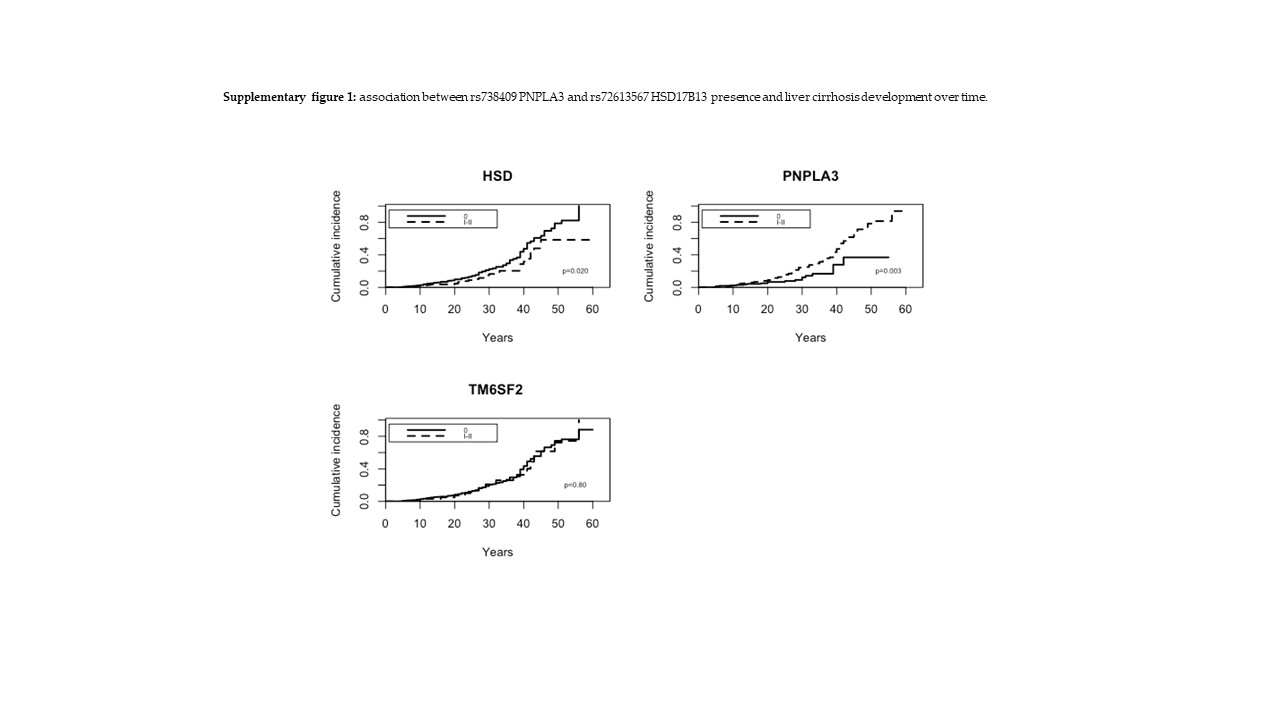

Supplement: Supplementary file 1 [file biomedicines-11-02132-s001.zip › Supplementary figure 1.jpg]

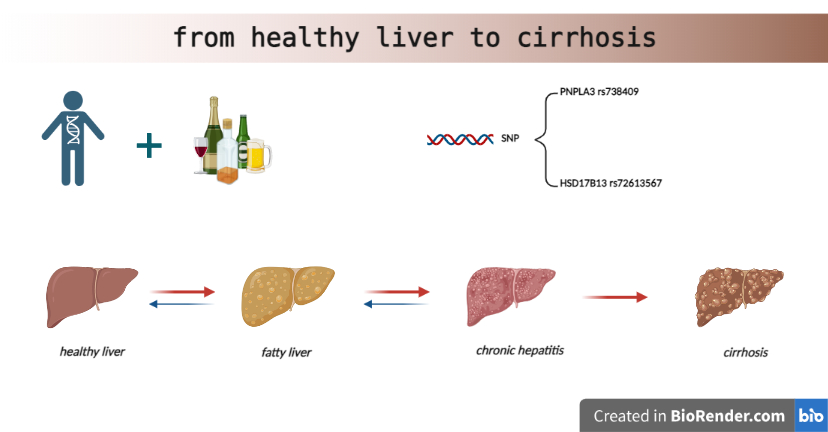

Supplement: Supplementary file 1 [file biomedicines-11-02132-s001.zip › Supplementary figure 2.jpg]
